# Beyond proteins: Ubiquitylation of lipopolysaccharide to fight bacteria

**DOI:** 10.1002/mco2.86

**Published:** 2021-09-09

**Authors:** Yanqiu Gong, Litong Nie, Lunzhi Dai

**Affiliations:** ^1^ State Key Laboratory of Biotherapy, National Clinical Research Center for Geriatrics and Department of General Practice, West China Hospital Sichuan University Chengdu China; ^2^ Department of Experimental Radiation Oncology MD Anderson Cancer Center, The University of Texas Houston Texas USA

## Abstract

Mechanisms and functions of protein ubiquitylation and LPS ubiquitylation. LPS ubiquitylation serves as a scaffold to recruit E3 ligases for the ubiquitylation of the membrane of bacteria. Polyubiquitin coat on the bacterial cell surface is one type of “eat‐me” signal recognized by the host cells. The abbreviations used in the figure are Pro, Protein; Ub, Ubiquitin; E1, Ubiquitin‐activating enzymes; E2, Ubiquitin‐conjugating enzymes; E3, Ubiquitin‐ligating enzymes; LPS, lipopolysaccharide; RNF213, Ring Finger Protein 213; and LUBAC, linear ubiquitin chain assembly complex.

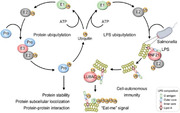

Polyubiquitylation on the membrane of bacteria is one type of “eat‐me” signal. However, the mechanism of forming the bacterial ubiquitin coat remains elusive. Recently, Otten et al. described the ubiquitylation of lipopolysaccharide (LPS), which can recruit E3 ligases to the bacteria to generate the ubiquitin coat.^1^ The discovery of LPS ubiquitylation expands the substrate scope and the functions of ubiquitylation.

Classical ubiquitylation, also known as ubiquitylation, formed by covalent attachment of ubiquitin(s) to the amino residues (or occasionally hydroxyl groups) of proteins to generate mono‐ or poly‐ubiquitylation, has essential roles in numerous cellular processes and pathways such as protein degradation,^2^ protein subcellular localization,^3^ and protein‐protein interaction.^4^ The protein ubiquitylation consists of a canonical three‐step enzymatic cascade driven by E1 (ubiquitin‐activating), E2 (ubiquitin‐conjugating), and E3 (ubiquitin‐ligating) enzymes^5^, ^6^ (Figure 1). Distinct from the consensus that ubiquitylation exclusively occurs on proteins, Otten et al. recently described non‐classical ubiquitylation that occurred on LPS upon bacterial infection.^1^

**FIGURE 1:**
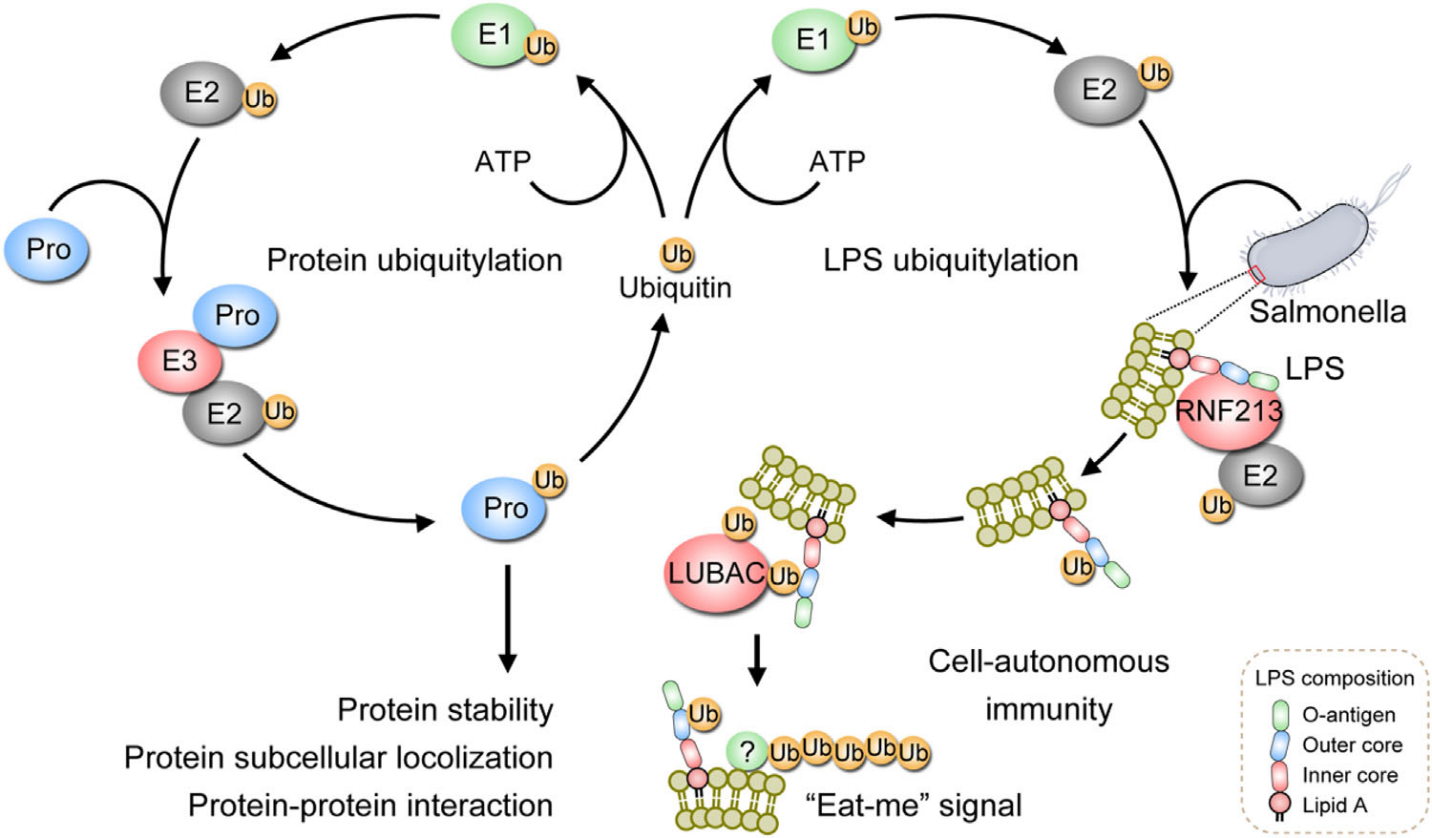
Mechanisms and functions of protein ubiquitylation and lipopolysaccharide (LPS) ubiquitylation. LPS ubiquitylation serves as a scaffold to recruit E3 ligases for the ubiquitylation of the membrane of bacteria. Polyubiquitin coat on the bacterial cell surface is one type of “eat‐me” signal recognized by the host cells. The abbreviations used in the figure: Pro, protein; Ub, ubiquitin; E1, ubiquitin‐activating enzymes; E2, ubiquitin‐conjugating enzymes; E3, ubiquitin‐ligating enzymes; LPS, lipopolysaccharide; RNF213, ring finger protein 213; LUBAC, linear ubiquitin chain assembly complex

Otten et al. isolated the wild‐type bacteria from infected cells and revealed a ubiquitin smear above 50 kDa with immunoblot. According to the size of the smallest band (15 kDa) of the Δ*rfc* mutant of *Salmonella Typhimurium* that contains oligo‐ubiquitylated substrate, they speculated the occurrence of LPS ubiquitylation based on the molecular weight of LPS (7 kDa) and ubiquitin (8 kDa). To confirm the hypothesis, they used a Bugbuster reagent which could maintain the native protein conformation to separate bacterial lysates. The disappearance of ubiquitin smear in higher molecular weights after denaturation was observed, while the 15 KDa band remained unchanged. To further verify the existence of ubiquitylated LPS, different mutants of *Salmonella* that generates diverse LPS structures were constructed. The band sizes imaged by immunoblots on ubiquitylated products matched the sizes of corresponding LPSs, demonstrating the ubiquitylation on LPS.

To reveal the enzymes of LPS ubiquitylation, Otten et al. applied sequential separation approaches followed by mass spectrometry. As a result, they identified the ring finger protein 213 (RNF213) of host cells as an E3 ligase, which was further confirmed by small interfering RNAs and CRISPR technology. RNF213 contains AAA+ domains in the dynein‐like core and RING domain in the C‐terminal E3 module.^7^ RING domain is usually responsible for protein ubiquitylation.^8^ However, by silencing the functions of certain domains, they revealed that RNF213‐mediated LPS ubiquitylation depended upon the catalytically active AAA+ module in the dynein‐like core rather than the RING domain. The zinc‐binding motif containing two conserved histidine residues and four conserved cysteine residues localized at the C‐terminal lobe was critical for RNF213‐mediated ubiquitylation of LPS, which might occur on the hydroxyl groups of sugars or fatty acids of lipid A, or on the phosphate groups of lipid A, or on both.

The ubiquitin coat on the bacterial cell surface generated by E3 ligases such as the linear ubiquitin chain assembly complex (LUBAC) is important for cell‐autonomous immunity.^9^ The formed ubiquitin coat can recruit proteins to activate immune responses and restrict the proliferation of bacteria via inducing autophagy, a cellular process that guides proteins, lipids, and/or organelles to lysosomes for degradation via autophagosome.^10^ How are these E3 ligases recruited to the bacteria to generate the ubiquitin coat? Otten and colleagues found that the LUBAC recruitment required RNF213‐mediated ubiquitylation of LPS localized at the outer membrane of the *Salmonella* surface (Figure 1). Depletion or mutations of RNF213 prevented the LUBAC recruitment and the formation of linear ubiquitin chains on the bacterial cell surface, consequently reducing the recruitment of ubiquitin‐binding autophagy receptors and the antibacterial autophagy of host cells, indicating the importance of pre‐existing ubiquitin on LPS for LUBAC recruitment.

The discovery of LPS ubiquitylation expands the substrate scope of ubiquitylation and the functions of the ubiquitylation system, and definitely directs future investigations in the area of ubiquitylation. It will be very interesting to characterize the ubiquitylation of many other non‐proteinaceous species such as lipids or carbohydrates. Chemical proteomics in combination with metabolomics and lipidomics methodologies probably serve as effective approaches for identifying the potential lipids or carbohydrates covalently linked to ubiquitin. The details of the mechanism of LPS ubiquitylation are still elusive. Many questions remain to be solved such as are there any other enzymes or proteins except RNF213 involving in LPS ubiquitylation, and are there any enzymes responsible for LPS deubiquitylation. LPS ubiquitylation may a general mechanism for host cells to fight against bacterial infection, which is required to be verified in other species of bacteria. Moreover, this work also suggests that enhancing cellular autophagy may be a good strategy for defending against bacterial infection. It will be very important to investigate whether those people with defects of LPS ubiquitylation‐related enzymes are susceptible individuals with bacterial infectious diseases.

## CONFLICT OF INTEREST

The authors declare that they have no competing interest.

## ETHICS APPROVAL

No ethical approval is required.

## AUTHOR CONTRIBUTIONS

L.D. revised the manuscript. Y.G. and L.N. drafted the manuscript.

## Data Availability

No additional data are included.
